# Role of EPS, Dispersant and Nutrients on the Microbial Response and MOS Formation in the Subarctic Northeast Atlantic

**DOI:** 10.3389/fmicb.2017.00676

**Published:** 2017-04-21

**Authors:** Laura Duran Suja, Stephen Summers, Tony Gutierrez

**Affiliations:** Institute of Mechanical, Process and Energy Engineering, School of Engineering and Physical Sciences, Heriot-Watt UniversityEdinburgh, UK

**Keywords:** marine oil snow (MOS), Faroe-shetland channel, hydrocarbon-degrading bacteria, Deepwater Horizon, crude oil, marine environment

## Abstract

In this study we report the formation of marine oil snow (MOS), its associated microbial community, the factors influencing its formation, and the microbial response to crude oil in surface waters of the Faroe-Shetland Channel (FSC). The FSC is a subarctic region that is hydrodynamically complex located in the northeast Atlantic where oil extraction is currently occurring and where exploration is likely to expand into its deeper waters (>500 m). A major oil spill in this region may mirror the aftermath that ensued following the Deepwater Horizon (DWH) blowout in the Gulf of Mexico, where the massive influx of Macondo crude oil triggered the formation of copious quantities of rapidly sinking MOS and successional blooms of opportunistic oil-degrading bacteria. In laboratory experiments, we simulated environmental conditions in sea surface waters of the FSC using water collected from this site during the winter of 2015. We demonstrated that the presence of dispersant triggers the formation of MOS, and that nutrient amendments magnify this. Illumina MiSeq sequencing revealed the enrichment on MOS of associated oil-degrading (*Cycloclasticus*, *Thalassolituus*, *Marinobacter*) and EPS-producing (*Halomonas*, *Pseudoalteromonas*, *Alteromonas*) bacteria, and included major representation by *Psychrobacter* and *Cobetia* with putative oil-degrading/EPS-producing qualities. The formation of marine snow, in the absence of crude oil and dispersant, in seawater amended with nutrients alone indicated that the *de novo* synthesis of bacterial EPS is a key factor in MOS formation, and the glycoprotein composition of the MOS aggregates confirmed that its amorphous biopolymeric matrix was of microbial (likely bacterial) origin. The presence of dispersants and crude oil with/without nutrients resulted in distinct microbial responses marked by intermittent, and in some cases short-lived, blooms of opportunistic heterotrophs, principally obligate hydrocarbonoclastic (*Alcanivorax*, *Cycloclasticus*, *Thalassolituus*, *Marinobacter*) and EPS-producing (*Halomonas*, *Alteromonas*, *Pseudoalteromonas*) bacteria. Interestingly, members of the *Vibrionales* (principally the genus *Vibrio*) were strongly enriched by crude oil (with/without dispersant or nutrients), highlighting a putative importance for these organisms in crude oil biodegradation in the FSC. Our findings mirror those observed at DWH and hence underscore their broad relevance.

## Introduction

A distinctive feature of the Deepwater Horizon (DWH) oil spill was the formation of large quantities of marine oil snow (MOS) that were observed in profuse quantities on the sea surface near and around the blowout (Passow et al., 2012). MOS was observed by scientists on the first research cruise that reached the spill site within 2 weeks from the onset of the spill on April 20 of 2010, and it sparked intense interest to understand the factors that triggered and influenced its genesis and evolution. MOS can be described as mucilaginous floating organic particles of >0.5 mm in diameter containing oil droplets embedded within its amorphous matrix. By June 2010, a little over a month after the onset of the spill, MOS was no longer visible at DWH and it is now understood that it had rapidly sedimented – a process termed MOSSFA (Marine Oil Snow Sedimentation and Flocculent Accumulation) – taking with it a significant fraction (ca. 14%) of the Macondo oil to the seafloor (Valentine et al., 2014) – it was found deposited east of the DWH spill site in the DeSoto Canyon region (Brooks et al., 2015) and further afield in the Gulf of Mexico (Valentine et al., 2014; Chanton et al., 2015; Romero et al., 2015).

It has been suggested that MOS formation may be a consequence of the interaction between oil and suspended organic matter, including dispersants (Fu et al., 2014; Kleindienst et al., 2015a) and eukaryotic phytoplankton cells (Passow, 2016). In roller-bottle experiments performed under conditions simulating sea surface conditions during the DWH spill, EPS produced by oil-degrading bacteria enriched in sea surface oil slicks was shown to trigger MOS formation (Gutierrez et al., 2013), and similar results were observed with EPS produced by axenic cultures of eukaryotic phytoplankton (van Eenennaam et al., 2016). Furthermore, Ziervogel et al. (2012) demonstrated that the suspended MOS particles acted as ‘hotspots’ for microbial oil-degrading activity, and Arnosti et al. (2016) showed MOS particles contained an associated bacterial community that was distinctly different from the free-living community in the surrounding seawater. Whilst the underlying mechanism(s) affecting the formation of MOS have yet to be properly understood, various factors, such as hydrodynamic conditions, collision rate of suspended particles, particle coagulation and flocculation and interaction of oil components with microorganism may be important in this process (Passow et al., 2012).

Considering that one of the largest reservoirs of organic matter on the Earth is found in the oceans, existing in the form of dissolved organic matter (DOM) – ca. 6.9 × 10^17^ g C, which is comparable in mass to the carbon in atmospheric CO_2_ (Hansell and Carlson, 2001) – its role in MOS formation has been a focus of many recent reports. DOM can play various functions and important roles in chemical, biological and physical oceanography, and is involved in fueling the microbial loop and in generating gasses (CO, CO_2_) and nutrients (Pomeroy, 1974; Azam et al., 1983). Much of this marine DOM is produced and released by bacteria and eukaryotic phytoplankton as extracellular polymeric substances (EPS) (Decho, 1990; Santschi et al., 1999). These are high molecular-weight polymers that are mainly composed of monosaccharides, but can also contain non-carbohydrate substituents (e.g., amino acid groups). A number of studies have reported large quantities of EPS in sea surface and deep-sea environments, including Antarctic marine waters (Mancuso Nichols et al., 2004 and references therein). Marine bacteria can contribute large quantities of EPS to the total DOM pool in the ocean (Azam, 1998), a large fraction of which exists as glycoproteins (Long and Azam, 1996; Verdugo et al., 2004). Since EPS, in particular those produced by marine bacteria, are endowed with an overall negative charge that is largely conferred by carboxyl groups of uronic acids (Kennedy and Sutherland, 1987), these acids have been implicated in the capacity of EPS to complex with metals (Gyurcsik and Nagy, 2000; Bhaskar and Bhosle, 2005). For example, at hydrothermal vents where these polymers are found, they can participate in complexing metal ions or radionuclides and contribute to their mobility and entry into the food web (Mancuso Nichols et al., 2004 and references therein). Some studies have also shown that uronic acids can confer EPS with an ability to interact with and increase the dissolution of hydrophobic organic chemicals, such as oil hydrocarbons (Janecka et al., 2002; Gutierrez et al., 2008, 2009). Amino acids and peptides, which are also often found associated with marine bacterial EPS, can also confer amphiphilic characteristics to these polymers and hence ability to interact with oils (Decho, 1990; Wolfaardt et al., 1999; Gutierrez et al., 2009).

Following a deep water oil spill, there are multiple stages of evolution for the oil, the first stage of which is rapid and concerns a non-hydrostatic release and adjustment of a buoyant plume from the wellhead. Uncontrolled and without intervention, the oil rapidly rises to become a surface slick in a matter of hours. Intervention with dispersants, however, can effectively emulsify a proportion of the oil and reduce its buoyancy, reminiscent to that during the DWH spill, which led to the formation of a massive subsurface oil plume at 1000–1300 m depth. With oil exploration expanding into more challenging environments, such as the Arctic and in deeper waters, it is necessary to instigate studies that aim to understand the fate of oil in these types of environments. One region of interest is the Faroe-Shetland Channel (FSC), which is located between the Faroe Islands (Faroese plateau) and the Shetland Islands (Scottish continental shelf) located ca. 100–150 miles north of Scotland’s most northerly mainland point. The FSC is a subarctic region defined by a dynamically complex confluence of different water masses, defined by active water mixing zones, variable physical conditions and large water masses flowing in opposite directions (Berx et al., 2013); it is the ‘spaghetti junction’ of Icelandic, Norwegian and Atlantic currents. Current oil extraction in the FSC occurs at depths down to ca. 500 m, though oil exploration may expand in the future to depths down to 1500 m. In the event of an oil spill in the FSC, the formation of MOS and its subsequent sedimentation to the seafloor by the process of MOSSFA could cause significant impacts to sensitive benthic ecosystems in these waters, such as rich communities of sponge fauna, the scleractinian coral *Lophelia pertusa*, polychaetes and anemones (Frederiksen et al., 1992; Howell, 2010).

Since the formation of MOS and how oil-degrading communities respond to oil contamination can differ substantially both in space and time in the global ocean, here we investigate MOS formation and the microbial community response to crude oil in surface waters of the FSC, and compare and contrast this to the Gulf of Mexico. We explore this using a deep-sequencing approach with the surface seawater treated with and without nutrient and dispersant amendments, and discuss the role of natural seawater EPS, dispersants and nutrients in influencing MOS formation in the FSC, and of the MOS-associated bacterial community. The findings of this work are anticipated to provide a greater level of understanding on MOS formation and the microbial community response in the FSC as a reference of a contrasting Atlantic water body to the Gulf of Mexico, and to help predict where the oil could end up on the seafloor in the event of an oil spill in this region.

## Materials and Methods

### Field Samples

**FIGURE 1:**
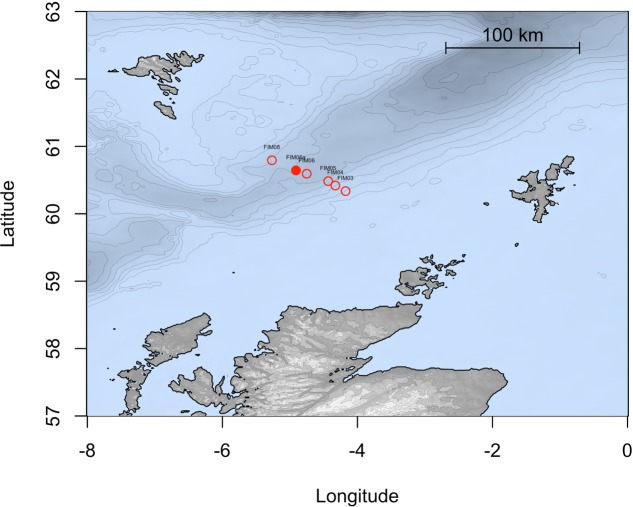
**Map representing the stations studied along the FIM section of the Faroe Shetland channel**. The solid point shows the station sampled in this study.

During a research cruise on the MRV *Scotia* on 15 December 2015, sea surface water samples were collected from a depth of 5 m in the FSC (60°38.12′ N, 4°54.03′ W; temp. 8.7°C) at approximately 10 km from the Schiehallion oilfield. Sampling was conducted along the Fair Isle-Munken (FIM) line, which is a sampling transect that runs between the Faroe and Shetland Isles. The water samples fall within a water mass defined as Modified North Atlantic Water (MNAW) which originates from the Faroe Islands and travels in a south to south-westerly direction through the FSC before diverging at the south end of the FSC (**Figure 1**). MNAW is a warm and saltier Atlantic water mass compared to the underlying Arctic/Icelandic cold-water masses that are found at depths from 400 to 1500 m in the FSC (Berx et al., 2013). The water samples were immediately stored at 4°C aboard the ship in 10 L carboys and used within 1 week for the preparation of water-accommodated fractions (WAFs) and in enrichment experiments with crude oil, dispersant and/or nutrient amendment.

### Water-accommodated Fractions

A WAF is defined as a laboratory-prepared medium containing dispersed and solubilized crude oil hydrocarbons/droplets by mixing a bulk liquid (e.g., seawater) with crude oil, and subsequent removal of the non-dispersed/solubilised oil. Here, WAF was prepared following the method of Kleindienst et al. (2015b). Briefly, seawater collected from the FSC was first passaged through 0.22 μm filters to remove microbial cells, with the exception that the filtrate was not pasteurized as described in the method of Kleindienst et al. (2015b). Heat treatment could alter seawater chemistry, in particular the molecular integrity of DOM, such as bacterial EPS which was found to play a direct role in MOS formation during the DWH oil spill (Gutierrez et al., 2013). A 100-mL volume of the filter-sterilized seawater was amended with 17.6 mL of pre-filtered (0.22 μm) Schiehallion crude oil (provided by BP) from the offshore Schiehallion oilfield located approximately 175 km west of the Shetland Isles. Seawater amended with only dispersant comprised 100 mL of the filter-sterile seawater and 1.76 mL of Superdispersant-25, which is a UK-approved dispersant. The effective dilution of the dispersant in seawater (dispersant-to-seawater ratio, v/v) was 1:10, which is a dilution that is recommended by the oil and gas industry (Approved oil spill treatment products, Government UK, July 2016). Chemically enhanced WAF (CEWAF) medium was prepared with 100 mL of sterile seawater amended with 17.6 mL of filtered Schiehallion crude oil and 1.76 mL of Superdispersant-25 – the effective dilution of the dispersant in this treatment was also 1:10.

The various mixtures of sterile seawater (SW) amended with oil (WAF), oil+dispersant (CEWAF) and solely dispersant (SW+D) were mixed on a rotary magnetic stirrer at 140 rpm for 48 h at 7°C in the dark in clean sterile (acid-washed) 500-mL glass bottles. The mixtures were allowed to stand for 1 h and then the aqueous phases (avoiding non-dispersed/solubilized oil or dispersant) were sub-sampled into clean (autoclaved and acid-washed with 5% nitric acid) screw-capped glass tubes with Teflon caps. These WAF, CEWAF and SW+D solutions were stored at 4**°**C and used within 48 h for the various microcosm experiments. Treatments containing nutrients – i.e., seawater+nutrients (SW+N) and CEWAF+nutrients (CEWAF+N) – were amended with 10 μM ammonium chloride, 10 μM sodium nitrate and 1 μM potassium phosphate (final concentrations).

### Microcosm Setup and Sampling

To examine the microbial response and formation of MOS in sea surface waters of the FSC when exposed to crude oil, dispersant and/or nutrients, a roller-bottle design was used as previously described (Gutierrez et al., 2013). For this, four microcosm experimental treatments were setup, each prepared using 250-ml Pyrexaaa glass bottles (38 × 265 mm) that were maintained in constant and gentle motion in order to simulate pelagic seawater conditions (Jackson, 1994). Each treatment was run in duplicate and comprised of 42.75 ml of filter-sterile WAF, dispersant-only, or CEWAF (with/without nutrients) added to 150 ml of unfiltered natural seawater from the FSC. In addition, two oil-dispersant untreated controls were setup and run in parallel: one comprised seawater alone with no other additions (SW), and the second of seawater with only nutrients added (SW+N). Treatments and controls were each established in duplicates and incubated at 7**°**C (approx. sea surface temp. in the FSC at the time of sampling) and in the dark at a rotation speed of 15 rpm. The treatments and controls were sampled at five time points over the course of 6 weeks: T_0_ at day 0, T_1_ after 1 week, T_2_ after 2.5 weeks, T_4_ after 4 weeks, and T_6_ after 6 weeks. At each sampling time, the bottles were placed in an upright position to capture a photographic record of MOS formation. Sub-samples of water were also withdrawn for DNA extraction and DAPI cell counts (see below); care was taken not to capture MOS particles in order to quantify bacterial abundance in the free-living fraction.

Visible aggregates were carefully withdrawn using glass Pasteur pipettes and transferred to 1.5-ml microcentrifuge tubes for staining with the cationic copper phthalocyanine dye alcian blue (AB) at pH 2.5 (Alldredge et al., 1993) or the amino acid-specific dye coomassie brilliant blue G (CBBG) at pH 7.4 (Long and Azam, 1996). AB is used for staining acidic sugars of EPS or transparent exopolymer particles (TEP) in seawater, whereas CBBG is used for staining the proteinaceous component of these polymeric substances. Following staining, the aggregates were washed by transferring them through several droplets of sterile water prior to their examination under the light microscope. To directly examine the prokaryotic community under the microscope, MOS particles were also stained with acridine orange (AO) (Francisc et al., 1973) for imaging with a FITC filter on a Zeiss Axioscope epifluorescence microscopy (Carl Zeiss, Germany). Moreover, the treatments and controls were observed daily over the course of the experiment for detection of any visual change, such as turbidity, emulsion and/or MOS formation.

### Genomic DNA Extraction

DNA was extracted from the original natural seawater collected from the FSC and from subsamples taken from each of the treatments and controls of the 6-week roller-bottle experiment. For this, ten milliliter samples were filtered using a glass column filtration system (Millipore) with 45 mm polycarbonate membrane filters (0.22 μm pore size; Isopore) and the filters stored at -20°C. The membrane filters were cut into three equal parts, and then each part placed into 1.5-mL microcentrifuge tubes and ground up with liquid nitrogen. The liquid nitrogen was allowed to completely evaporate from each tube and the contents extracted according to the method of Tillett and Neilan (2000). Purified DNA was stored at -20°C for subsequent molecular analysis.

### Barcoded Amplicon Metagenomic Sequencing and Analysis

Barcoded 16S rRNA gene MiSeq sequencing, targeting the V3-V4 hypervariable region, was employed to analyze the bacterial community over the 6-weeks duration of the experiments at time points T_0_, T_2_, T_4_ and T_6_. We amplified the 16S rRNA gene in duplicate 50 μl reactions. Each reaction comprised 32 μl molecular biology grade water, 10 μl 5x MyTaq polymerase reaction buffer, 2.5 μl 4 uM primer mix, 0.5 μl MyTaq Enzyme (2.5U; BioLine), 3 μl DMSO (6%), and 2 μl gDNA. The primers used were 341f-CCTACGGGNGGCWGCAG and 785r-GGACTACHVGGGTWTCTAAT. Both primers also had Illumina MiSeq overhangs attached to their 5′ ends. Barcodes were not added at this point of PCR. Thermocycler conditions for this first round of PCR were an initial denaturation of 96°C for 1 min, 32 cycles of 96°C for 15 sec, 55°C for 15 sec, and 72°C for 30s, and a final extension at 72°C for 3 min. PCR clean-up was performed using 20 μl of the PCR product, and adding 1 μl FastAP (1U), 0.5 μl Exonuclease I (10U; both Thermofisher) and 3.5 μl molecular grade water. Conditions for the PCR clean-up reaction were 45 min at 37°C followed by 15 min enzyme denaturation step at 85°C. The purified PCR amplicons were then subjected to a second-step PCR at the sequencing facility for the addition of the Golay barcodes, which were unique to each treatment.

All samples were sequenced via the Illumina MiSeq platform (Illumina 2 × 250 V.2 kit) at the University of Liverpool Centre for Genomic Research^1^; sequences were demultiplexed prior to receipt at our laboratory. Subsequent processing was conducted using the MiSeq SOP (accessed: September 2016) cited within the MOTHUR program (Kozich et al., 2013). In brief, single end reads were examined using MOTHUR (v1.36.1). Contiguous sequences were constructed from paired end sample reads. All sequences with any ambiguities or homopolymers longer than eight bases were excluded from further analysis. All remaining sequences were aligned against a SILVA compatible database. All sequences were trimmed to a maximum length of 465 bases before chimeric sequences were identified and removed using UCHIME (Edgar et al., 2011). The taxonomic identity of sequences was determined by comparison to a MOTHUR formatted RDP database (v.14). Any sequence returned as unknown, chloroplast or mitochondrial were removed from further downstream analysis. Operational taxonomic units (OTUs) were clustered based on 97% sequence identity and subsampled to 35,000 sequences per sample to eliminate sampling bias during subsequent diversity examination. All sequences were deposited in SRA repository under accession number SAMN06246901.

### Prokaryotic Cell Counts

To quantify prokaryotic (bacteria and archaea) cell counts, we used the DAPI (4′6-diamidino-2-phenylindole) staining technique. For this, sub-samples of water from each treatment and control of the roller-bottle incubations were fixed with 3.7% formaldehyde and stored at 4°C for a maximum of 2 weeks. The collection of MOS particles, as part of these water samples, was avoided here, as the accurate enumeration of cells associated with MOS was not feasible due to oil autofluorescence and obscured visualization of cells due to the agglomerate matrix. For each fixed water sample, 5 ml was filtered (0.22 μm) onto gridded (3 mm × 3 mm) polycarbonate filters – this volume was adjusted in order to achieve 10–150 cells per grid. The filters were mounted onto glass slides and the cells stained with DAPI (1 μg/ml) for 20 min and then counted under the Zeiss Axioscope epifluorescence microscope (Carl Zeiss, Germany). A minimum of 10 grids were randomly selected and photographed for counting of cells. The number of cells counted was calculated using the formula: *N* = (*n*_b_/*n*_Sq_) ×*V*_f_ × (*A*/*A*_Sq_), where *N* is the total number of bacteria per mL, *n*_b_ is the number of bacteria counted, *n*_Sq_ is the number of squares counted, *V*_f_ is the volume of sea water filtered, *A* is the effective filter area, and *A*_Sq_ is the area of one square of the grid.

### Statistical Analyses

Relative abundances of sequences obtained using MiSeq were compared using an NMDS plot to visualize β-diversities of each sample for both treatment and time point. An ANOSIM analysis was conducted to determine if there was any significant difference between treatments employed. Further examination of the α-diversity was achieved by generation of rarefaction curves, based on 97% sequence similarity. Moreover, Shannon-Weiner diversity indices of H’ were generated and compared using an analysis of variance to determine significant differences between diversity of treatments and time points sampled. All data was log transformed to meet the assumptions of parametric analysis.

## Results

### MOS Formation

**FIGURE 2:**
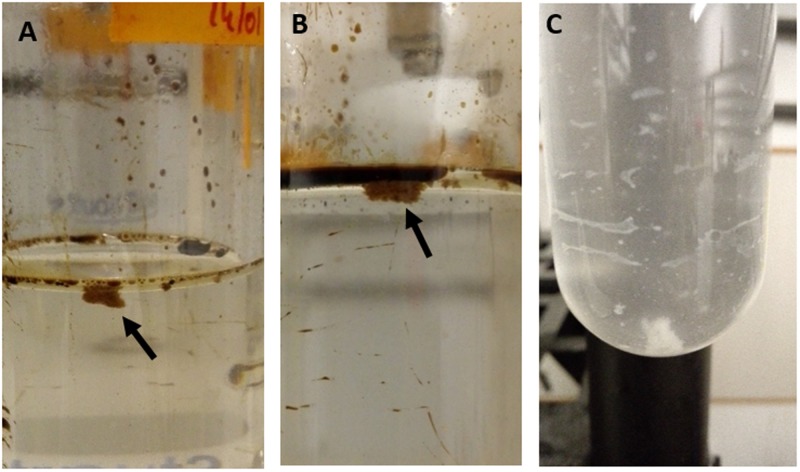
**Marine oil snow aggregates shown floating at the surface of the CEWAF (A)** and CEWAF+N **(B)** roller-bottle incubations, and Marine Dispersant Snow (MDS) aggregates shown settled at the bottom of the bottles in the SW+D treatments **(C)**.

In the roller-bottle microcosm incubations, a rapid formation of MOS was observed within 5 days in the CEWAF+N treatment, and within 7 days in the CEWAF treatment. In both treatments the MOS particles appeared brownish, round and of ‘fluffy’ texture (**Figures 2A,B**). Initially, the aggregates were small (<3 mm in diameter) and exhibiting amorphous definition. With the naked eye, small oil droplets could be seen associated within the amorphous matrix of the MOS aggregates from these treatments. Over the course of these roller-bottle incubations, the aggregates were observed to become progressively less buoyant, and by week 6 they settled to the bottom of the glass tubes when held in an upright position. The size of the MOS aggregates in these incubations (CEWAF and CEWAF+N) also increased over time (from initially 2–3 mm to ∼2 cm after 4 weeks) and we posit that smaller aggregates had merged together since we observed that the absolute abundance of MOS particles (i.e., that could be counted by visual observation) had decreased over time. By week 4, aggregate size appeared to stabilize (1–2 cm average aggregate size) and remained unchanged thereafter.

In the treatment of seawater with only dispersant (SW+D), however, the formation of large (size range 0.5–3 cm) white aggregates occurred within 3 days (**Figure 2C**), for which we propose to define hereafter as ‘marine dispersant snow’ (MDS). Aggregates of MDS were approximately 2–3 times larger in size compared to MOS aggregates that formed in the CEWAF and CEWAF+N treatments. As similarly observed for MOS particles in these treatments, MDS aggregates progressively lost their buoyancy and eventually, by week 2, settled to the bottom of the glass tubes when held in an upright position (**Figure 2C**). Manipulation of selected MDS aggregates on a microscope slide revealed they exhibited quite viscous/gelatinous characteristics.

In contrast, the formation of MOS was not observed in the WAF treatment, and no marine snow particles formed in the SW control incubations. However, in the SW+N treatment the formation of marine snow (no oil) was observed and these particles were comparatively small (1–2 mm) and remained so for the duration of these experiments. They were also observed to be extremely fragile and disintegrate when the incubation chamber was gently shaken.

**FIGURE 3:**
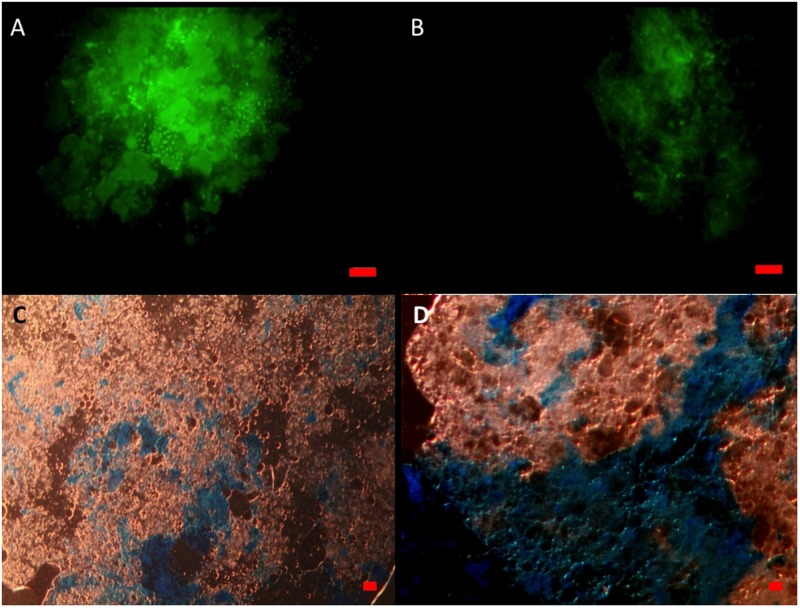
**Formation of MOS and marine snow in the roller bottle incubations**. Under the epifluorenscence microscope after staining with acridine orange, MOS **(A)** which formed in the CEWAF (±nutrients) treatments was populated with associated prokaryotic cells (small green dots) and oil droplets (larger green spherical/irregular blobs). Marine snow **(B)** that formed in the SW+N treatments contained few associated prokaryotic cells. Under the light microscope, MOS stained with coomassie brilliant blue G **(C)** and Alcian Blue **(D)**. Bar, 10 μm.

When viewed under the epifluorescence microscope with AO staining, MOS aggregates from the CEWAF and CEWAF+N treatments appeared as amorphous ‘fluffy’ particles with associated oil droplets (large green blobs; average size range 5 to >20 μm diameter) and represented foci for the attachment of prokaryotic cells (**Figure 3A**). Similarly, MDS aggregates also showed the presence of associated prokaryotic cells (results not shown). Marine snow particles (without oil) that formed in the SW+N treatment, however, were observed to contain markedly fewer prokaryotic cells (**Figure 3B**). When viewed under the light microscope with the aid of dark field illumination, MOS aggregates partially stained with CBBG (**Figure 3C**) and AB (**Figure 3D**).

### Bacterial Community Composition of MOS

Barcoded 16S rRNA Illumina MiSeq technology was used to analyze the bacterial community associated with MOS and of that in the surrounding (not associated with MOS) seawater. We were limited to performing this with only the CEWAF+N treatment because MOS aggregates that formed in this treatment maintained there structural integrity and did not disintegrate when handled; MOS aggregates from the other treatments were found to be quite fragile and handling them during the initial processing steps for MiSeq sequencing resulted in them breaking up and completely disintegrating.

**FIGURE 4:**
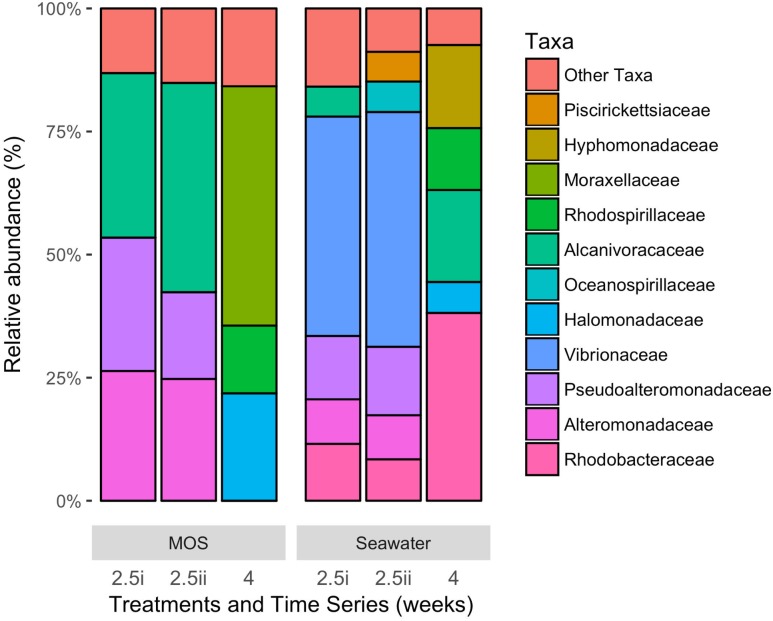
**Bacterial community composition at family-level classification of MOS compared to that in the surrounding seawater in the CEWAF+N treatment at weeks 2.5 and 4**.

**Figure 4** shows the bacterial community structure – at family-level classification – of MOS formed in the CEWAF+N treatment at weeks 2.5–4, presented here alongside the community of the surrounding seawater from the same roller-bottle incubation. Of a total of up to 448,754 high quality partial 16S rRNA gene sequences, the MOS bacterial community at the 2.5-week time point showed a clear dominance of members within the *Alcanivoracaceae*, *Alteromonadaceae* and *Pseudoalteromonadaceae* – respectively, on average 38.0, 25.5 and 22.4% of the total MOS-associated community. Minor representation (of >1%) included phyla within the *Rhodobacteraceae* (2.9%), *Rhodospirillaceae* (2.3%), *Vibrionaceae* (2.1%) and *Piscirickettsiaceae* (1.2%). In contrast, the bacterial community of the seawater surrounding the MOS aggregates from this same CEWAF+N treatment at week 2.5 was dominated by phyla within the *Vibrionaceae* (46.1%), with high contributions also by *Pseudoalteromonadaceae* (13.4%), *Rhodobacteraceae* (10.0%), *Alteromonadaceae* (9.0%), *Oceanospirillaceae* (5.6%), *Piscirickettsiaceae* (5.5%) and *Alcanivoracaceae* (5.3%).

An analysis of the major orders at the level of genus revealed some interesting groups that dominated the community associated with MOS in the CEWAF+N treatment when analyzed at the T_2_ and T_4_ time points compared to that in the surrounding seawater (**Supplementary Table S1**). At T_2_, MOS was dominated by members of the genera *Alcanivorax* (33–42%), *Pseudoalteromonas* (17–27% of total sequence reads), *Alteromonas* (25%), with minor representation by *Sulfitobacter*, *Vibrio*, *Thalassospira*, *Cycloclasticus* and *Mesonia* (collectively contributing < 10% of total reads). At the T_4_ time point, MOS was dominated by members of the genera *Psychrobacter* (48.4% of total sequence reads), *Cobetia* (21.6%), *Thalassospira* (13.8%), with minor representation by *Pseudoalteromonas* (4.4%), *Alcanivorax* (2.7%), *Cycloclasticus* (1.6%) and *Marinobacter* (1.2%). In terms of the number of 16S rRNA reads that were found enriched on MOS compared to their abundance in the surrounding seawater at the T_4_ time point, *Psychrobacter* was 970-fold higher in abundance, *Marinobacter* 20-fold higher, *Halomonas* 8.5-fold higher, *Pseudoalteromonas* 7.5-fold higher, *Cobetia*, *Cycloclasticus* and *Vibrio* 3.5-fold higher, *Alteromonas* 3-fold and *Thalassolituus* 1.5-fold higher.

### Bacterial Community Dynamics in the Various Treatments

**FIGURE 5:**
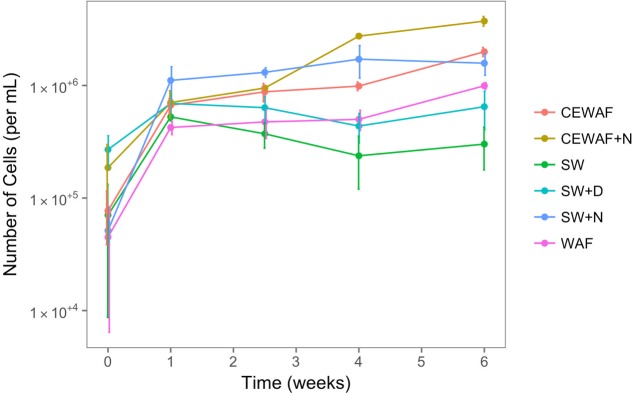
**Prokaryotic (bacterial and archaeal) cell numbers from roller-bottle incubations of the different treatments with sea surface water from the FSC amended with or without nutrients, dispersant and/or crude oil (as WAF)**. SW, seawater; SW+N, seawater with nutrients; SW+D, seawater with dispersant; WAF, water-accomodated fraction; CEWAF, chemically enhanced WAF; CEWAF+N, chemically enhanced WAF with nutrients.

To assess the free-living (not associated with MOS) prokaryotic community dynamics in the different treatments amended with and without nutrients, dispersant or crude oil, DAPI counts were determined over the 6-week duration of these experiments at time-points T_0_ (start day of the experiment), T_1_ (after 1 week), T_2_ (after 2.5 weeks), T_4_ (after 4 weeks) and T_6_ (after 6 weeks). As shown in **Figure 5**, prokaryotic cell abundance across all six treatments at the start of the experiment (T_0_) was 0.8–15.0 × 10^4^ cells/ml, and as expected cell abundance in the untreated control (SW) remained low relative to the other treatments throughout the 6-week duration of these experiments. Similarly, low prokaryotic cell abundances were achieved in the SW+D and WAF treatments (6.9 × 10^5^ and 9.9 × 10^5^ cells/ml, respectively). In the SW+N treatment, however, cell numbers showed the highest increase within the 1st week, and then slowing down to a steady increase over the proceeding 3 weeks, and reaching maximal abundances by week 4 (1.7 × 10^6^ cells/ml). Prokaryotic cell abundances in the CEWAF and CEWAF+N treatments followed a similar increasing trend initially, and their dynamics diverged after about 2 weeks. Cell abundances in the CEWAF+N treatment showed the most notable increase compared to the other treatments, reaching 3.7 × 10^6^ cells/ml by week 6.

We note that although our DAPI counts demonstrated an expected pattern for prokaryotic dynamics in these treatments, the counts are likely to be somewhat underestimated with particularly the treatments where MOS had formed due to the high numbers of DAPI-stained prokaryotic cells associated with MOS particles (**Figure 3**). The enumeration of the cells was practically impossible to count accurately because of their density and localization within and on the surface of the MOS aggregates.

**FIGURE 6:**
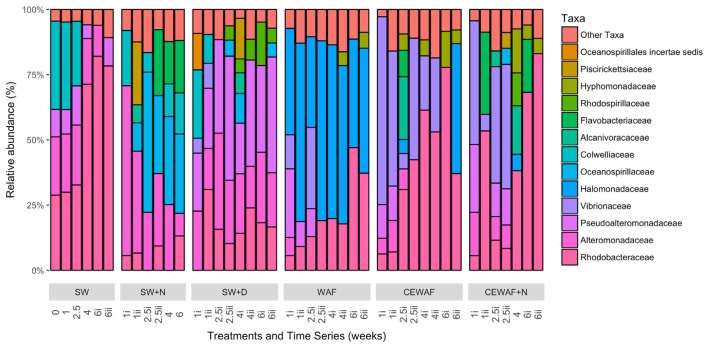
**Bacterial community composition at family level classification for each of the different treatments over the 6 week incubation period that the roller-bottle experiments were run**. SW, seawater; SW+N, seawater with nutrients; SW+D, seawater with dispersant; WAF, water-accomodated fraction; CEWAF, chemically enhanced WAF; CEWAF+N, chemically enhanced WAF with nutrients.

**FIGURE 7:**
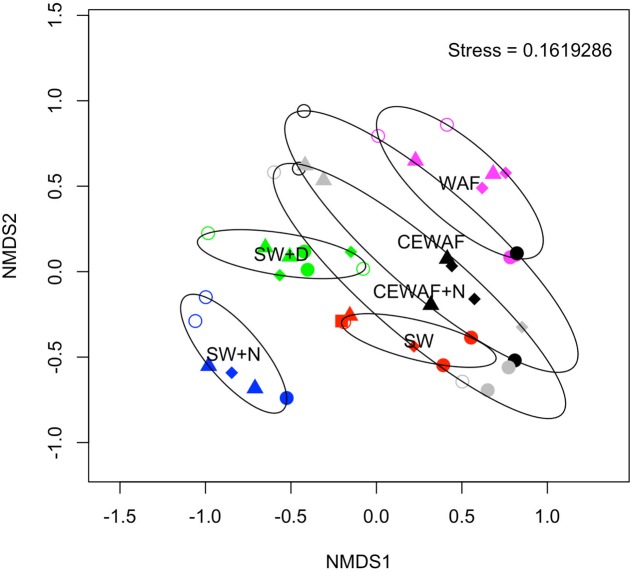
**Non-metric multidimensional scaling (NMDS) plot showing the similarity of each sample**. The stress achieved is indicated in the top right of the plot. Symbol colors signify the original treatment: SW (red); SW+N (blue); SW+D (green); WAF (purple); CEWAF (black); CEWAF+N (gray). Each time point is represented by a different symbol: T_0_ (square); T_1_ (open circle); T_2_ (triangle); T_4_ (diamond); T_6_ (closed circle). Ellipses shown surrounding symbols represent grouping the treatment types with 95% confidence interval.

The diversity of the bacterial communities in the surface seawater of the FSC and their response to and dynamics in the various treatments (SW, SW+N, SW+D, WAF, CEWAF, CEWAF+N) was assessed using Illumina MiSeq technology and shown at family-level classification in **Figure 6**. At the commencement of these experiments (denoted by time point T_0_), the community was initially dominated by groups within the *Alteromonadales* and *Rhodobacterales* – collectively 96% of total sequence reads. The major genera of this T_0_ community constituted *Colwellia* (33.7%), *Sulfitobacter* (28.2%), *Pseudoalteromonas* (10.5%), *Alteromonas* (2.7%) and other members of the family *Alteromonadaceae* (22.4%) (**Supplementary Table S1**). Other phyla, such as the hydrocarbonoclastic bacteria *Alcanivorax*, *Cycloclasticus*, *Marinobacter* and *Thalassolituus*, as well as *Halomonas* that, like *Alteromonas* and *Pseudoalteromonas*, are recognized producers of EPS, were also present though in low abundance (≤0.5% for each; **Supplementary Table S1**). This bacterial community of the FSC sea surface in the untreated control (SW) maintained a relatively consistent structure throughout the 6-week duration of these experiments. Rarefaction analysis of a sub-set (35,000 sequences) of the 16S rRNA gene sequences showed that for no treatment was saturation of sequencing reached (**Supplementary Figure S1**). The OTU richness of each treatment ranged between 520 and 1,010 of identified OTUs at T_1_, and upon the termination of the experiment (T_6_) all the treatments exhibited, with the exception of SW+N and CEWAF+N, a reduction in the number of OTUs. Overall the α-diversity indices (Shannon-Weiner H’) for each treatment indicated that only SW+D and SW+N had higher diversities than were measured for the SW controls (**Supplementary Figure S2A**; ANOVA, *F*_5_ = 0.05326, *p* < 0.01). Moreover, diversity also declined overall during the period of the experiment (**Supplementary Figure S2B**; ANOVA, *F*_1_ = 0.1192, *p* < 0.01). The similarity between treatments and samples therein can be visualized in **Figure 7**. This indicated the similarity of each sample to all other samples examined and confirmed that there was significant dissimilarity between the bacterial communities within the treatments (ANOSIM, *R* = 0.6624, *p* < 0.001). Here it can be observed that the β-diversity most prominently differs between water types and not time points measured. Most distinct is the SW+N and SW+D treatments.

Within the first week, this complex community of the FSC surface seawater became overlayed by opportunistic bacteria that were stimulated by the presence of either nutrients, dispersant and/or crude oil. The community in the SW+N control treatment showed a rapid enrichment of members within the order *Alteromonadales*, mainly of the genus *Alteromonas*, which remained as a dominant group (21–60%) until the termination of the experiment when their abundance decreased to ca. 8% at week 6. To a lesser extent, *Neptuniibacter* within the order *Oceanospirillales* and members of the family *Flavobacteriaceae* were also dominant groups that bloomed by week 2 and collectively persisted as the most dominant groups for the remaining duration of these incubations. Furthermore, a progressive enrichment of members within the *Rhodobacteraceae* occurred by week 1 and reached 13% of the total community by week 6. Similarly in the SW+D treatment, members of the *Alteromonadales* dominated the community, though this time contributed by the genera *Pseudoalteromonas* and *Alteromonas*, and their dominance persisted for the remaining duration of the experiment. However, the community showed clear, though lower, representation of groups within the *Rhodospirillales*, mainly contributed by the genus *Thalassospira*, and by members of the *Rhodobacterales* and *Oceanospirillales*, and by phyla of the class *Gammaproteobacteria* that included the genus *Vibrio*, although it bloomed (up to 10%) in only the first week. Compared to the SW and SW+N controls, the presence of dispersant in the SW+D treatment effected a clear reduction (to <1%) in the abundance of *Colwellia* by week 2.

In the WAF treatment, where the FSC seawater was amended with crude oil in the form of a WAF, the microbial response was distinctly different compared to the SW, SW+N and SW+D treatment. Within 1 week the community of the WAF treatment became strongly dominated by members of the *Oceanospirillales*, largely of the genus *Halomonas* (34–65%) that persisted as the major group for the remaining duration of the experiment. Other major groups included members of the *Alteromonodales*, largely of the genus *Pseudoalteromonas* but that bloomed in week 1 (up to 25%) and progressively decreased in abundance thereafter. Members of the family *Rhodobacteraceae* increased in abundance from week 1, reaching maximal levels (42.2%) by week 6, and a short-lived bloom of *Vibrio* occurred within weeks 1–2. As observed in the SW+D treatment, the abundance of *Colwellia* dramatically decreased in abundance, this time within week 1, in the WAF treatment.

The bacterial community response in the CEWAF and CEWAF+N treatments contrasted substantially to the controls and other treatments. Here, the bacterial community response to the oil and dispersant (in the presence or absence of nutrients) revealed a more complex pattern of microbial succession, especially when viewed at the genus-level classification, over the 6-week duration of these incubations. Within the first week, both treatments showed a strong bloom for members of the *Vibrionales*, principally the genus *Vibrio*, that then decreased in abundance by week 3 in the CEWAF+N treatment, and by week 4 in the CEWAF treatment. In parallel, a progressive decrease in the abundance for members of the order *Alteromonadales* occurred within the first few weeks, principally contributed by the genera *Colwellia*. As similarly observed in the SW+D and WAF treatments, the abundance of *Colwellia* in these CEWAF and CEWAF+N treatments dramatically decreased within week 1 and remained in very low abundance (<0.4%) for the remaining duration of these experiments. On the other hand, *Pseudoalteromonas*, also a member of the order *Alteromonadales*, remained at relatively high abundance for the first week in the CEWAF treatment (13.2%) and second week in the CEWAF+N treatment (13.4–14.1%) before decreasing thereafter, whereas *Marinobacter* bloomed intermittently at weeks 1–2 in, respectively, the CEWAF (6.5%) and CEWAF+N (6.2%) treatments. Other phyla that became strongly selected for in these oil-dispersant treatments were members of the order *Rhodobacterales* within the class *Alphaproteobacteria*, principally contributed by *Sulfitobacter* in only the CEWAF treatment, and other members of the family *Rhodobacteraceae* in both treatments, with minor representation by *Hyphomonas* at week 6 in the CEWAF treatment and in weeks 4–6 in the CEWAF+N treatment. Other enriched groups of *Rhodobacterales* included *Oceanicaulis* and *Oceanicola* in only the CEWAF treatment at weeks 2–6. Short-lived blooms of *Mesonia*, of the class *Bacteroidetes*, occurred at week 2 and in weeks 4–6 in, respectively, the CEWAF and CEWAF+N treatments.

Notably, the enrichment of the obligate alkane-degrader *Alcanivorax* occurred in the CEWAF treatment during the first 2 weeks and which was prolonged into week 4 in the CEWAF+N treatment before the abundance of these organisms dissipated thereafter in both of these treatments to background levels. Members of another alkane-degrader, *Thalassolituus*, and of the PAH-degrader *Cycloclasticus* were also enriched in only the CEWAF+N treatment, occurring during week 2. A less pronounced enrichment of members within the genus *Thalassospira*, for which some species have been described to degrade hydrocarbons, occurred in both these treatments and only toward the end of these incubations.

## Discussion

To our knowledge this is the first study examining the formation of MOS in northeast Atlantic waters. We specifically focused on the FSC where subsurface oil extraction is currently occurring and where exploration for oil in deeper waters (>1000 m depth) within this channel may expand in the near future. This is important given that an oil spill in the deep waters of the FSC could produce a similar oil spill as occurred during the DWH blowout in the Gulf of Mexico, and one that could be considerably more complex and difficult to combat given how much more hydrodynamic the FSC water column is compared to the Gulf of Mexico. Our findings showed that crude oil alone does not act as an inducer for MOS formation in surface waters of the FSC, and that the addition of dispersant in the presence of oil appeared to be an important factor in triggering MOS formation, as observed in the CEWAF and CEWAF+N treatments. Even in the absence of crude oil, however, aggregates formed in the SW+D treatment and resembled those observed in the experiments of Kleindienst et al. (2015b) who used water from the Gulf of Mexico supplemented with the dispersant Corexit – a dispersant that was profusely used by BP on sea surface oil slicks and pumped directly at the leaky Macondo well-head during the DWH spill (National-Commission on the BP Deepwater Horizon Oil Spill and Offshore Drilling, 2011). In both studies, these dispersant aggregates appeared white, gelatinous and viscous when handled. These findings suggest that contrasting waters – i.e., the Gulf of Mexico and FSC – can lead to the formation of dispersant-induced aggregates displaying similar macroscopic characteristics. Since the use of dispersants in a marine setting is mainly as a contingency to combat oil spills, the SW+D treatment acted as a semi-control to test the effects of the dispersant on the microbial community in our experiments and is discussed below.

The formation of MOS is likened to marine snow particles that are a crucial component of the biological pump in the ocean and defined as ‘hot spots’ for microbial activity (Long and Azam, 2001; Daly et al., 2016) where their exists a heightened level of enzyme activity and degradation rates compared to that in the seawater environment immediately surrounding these particles (Smith et al., 1992). As reported by Giani et al. (2005), the formation of marine snow correlates with specific physical conditions (water column stratification, low mixing) and biological production patterns in the water column, such as nutrient concentrations, microbial production of TEP and EPS. Furthermore, the formation and evolution of marine snow particles can vary considerably in terms of their size and content of mucilaginous matter, such as TEP and EPS (Giani et al., 2005; Passow et al., 2012) which are a matrix for marine snow formation (Passow, 2016). Our results showed that the presence of dispersant and crude oil (CEWAF treatment) yields MOS, but that oil alone (WAF treatment) does not. Furthermore, the addition of nutrients alone to seawater (SW+N treatment) triggers the formation of marine snow in surface waters of the FSC. Notably, nutrients amplified the abundance and size of MOS particles, as observed in the CEWAF+N treatment, and the structural integrity of these nutrient-aided MOS particles was more robust compared to that of their counterparts formed in the CEWAF treatment without added nutrients. van Eenennaam et al. (2016) also described the formation of fragile MOS that easily falls apart when agitated, and that the bacteria associated with eukaryotic phytoplankton, principally through their production and release of EPS, enhances MOS formation. These findings indicate that microorganisms, in particular EPS-producing bacteria, play a key role in MOS formation, and that nutrients enhanced the activities of these organisms and yielded higher concentrations of EPS in the CEWAF+N. We hypothesize that EPS then interacted with crude oil and/or dispersant to form MOS, as previously observed (Gutierrez et al., 2013). Other reports have also shown nutrient additions to seawater in influencing the formation of MOS (Kleindienst et al., 2015b; Daly et al., 2016 and references therein).

We used MiSeq sequencing to examine the bacterial taxa that were influenced by nutrients and potentially induced or upregulated the release of EPS and effected MOS formation in the CEWAF+N treatment. Hitherto, the only published study to have examined this type of community analysis for MOS was reported by Arnosti et al. (2016) who used Sanger sequencing of clone libraries to analyze the bacterial community associated with MOS that formed in roller-bottle experiments with sea surface waters from the Gulf of Mexico. As in our present study, Arnosti et al. (2016) showed their MOS aggregates harbored a bacterial community composition that was distinctly different from that in the surrounding seawater. The MOS aggregates from the Gulf of Mexico were primarily composed of oil-degrading (*Cycloclasticus*, *Marinobacter*) and EPS-producing (*Halomonas*) bacteria, including diverse members of the order *Rhodobacterales* (principally within the family *Rhodobacteraceae*). This corroborates our results with FSC surface waters where we identified the enrichment of these taxa on MOS formed in the CEWAF+N treatment, as well as other taxa with recognized oil-degrading (*Alcanivorax*, *Thalassolituus*, *Thalassospira*) and EPS-producing (*Pseudoalteromonas*, *Alteromonas*, *Vibrio*, *Cobetia*) qualities. This enrichment of bacterial taxa which specialize in oil-degradation and EPS production is consistent with the reduction in α-diversity observed in the water fractions of this study over the course of the incubations (**Supplementary Figure S2B**). Kleindienst et al. (2015b) used catalyzed reporter deposition in combination with fluorescence *in situ* hybridization (CARD–FISH) to analyze MOS aggregates from their CEWAF+N treatments with Gulf of Mexico seawater and found the aggregates were dominated by members of the class *Gammaproteobacteria*, including the order *Alteromonadales*, and in particular members of the genus *Colwellia*, hence suggesting that *Colwellia* may play an important role in MOS formation in the presence of dispersants. During incubations with uncontaminated deep water samples collected during the active phase of the Gulf oil spill, Baelum et al. (2012) also reported the formation of MOS which was also dominated by members most closely related to *Colwellia*. Conversely, *Colwellia* contributed 0.01% abundance to MOS that formed in our CEWAF+N treatments by week 4 (T_4_), and the abundance of these organisms decreased sharply within week 1 in all the treatments amended with dispersant and/or crude oil. Hence, these contrasting water bodies of the Atlantic region (i.e., the Gulf of Mexico vs FSC) differ with respect to the bacterial taxa associated with MOS, and potentially also its formation and fate.

Interestingly, the most dominant organisms associated with MOS were members of the genus *Psychrobacter* (48.5% of total community reads from T_4_ samples), which is a genus recognized for cold-tolerance – some have been isolated from permafrost and Antarctic waters – and reported to produce EPS (Leiye et al., 2016). These organisms have also been found in waters contaminated with crude oil in the Arctic (Deppe et al., 2005) and Antarctic Sea (Prabagaran et al., 2007), hence suggesting putative hydrocarbon-degrading qualities. *Psychrobacter* has not previously been reported associated with MOS, and based on its dominant abundance of the total MOS-associated bacterial community these organisms may be an important contributor to MOS formation in surface waters of the FSC and/or the degradation of oil droplets associated with these aggregates. Although, further work will be needed to better elucidate this. We posit that the collective community of opportunistic heterotrophs associated with MOS contributes two key roles. The first is in the formation of MOS, which we hypothesize is mediated by EPS of organisms such as *Pseudoalteromonas* and *Alteromonas* that were abundant taxa associated with MOS at the initial stages of its formation (T_2_) in our experiments. The second is to the degradation of hydrocarbons within crude oil droplets entrained within the amorphous ‘net-like’ scaffolding of MOS. We hypothesize that hydrocarbon-degradation rates are markedly higher on MOS aggregates compared to in the surrounding seawater medium. This is supported by high rates of lipase hydrolysis acitivity detected on MOS aggregates formed in roller bottle experiments with surface seawater collected from the Gulf fo Mexico during the DWH oil spill (Ziervogel et al., 2012). The enrichment of obligate hydrocarbonoclastic bacteria on MOS, such as members of the genus *Alcanivorax* (33–42% relative abundance of the total MOS community) identified in our experiments, indicates MOS as a niche environment where oil biodegradation activities may be significantly elevated compared to that in the surrounding seawater environment. By week 4 the *Alcanivorax* population associated with MOS had decreased to <3% of the total community, suggesting that the bulk of the *n*-alkane hydrocarbons, which these organisms preferentially use as carbon substrates, had become sufficiently depleted on the MOS aggregates. This assumes these organisms had detached from the MOS aggregates to find new sources of utilizable hydrocarbons, which corroborates with the observed increase in their relative abundance in the seawater environment surrounding these aggregates.

Considering that MOS had already been observed on surface waters of the Gulf of Mexico during the DWH oil spill before BP had begun their operation of spraying tons of dispersants (Passow et al., 2012), and laboratory roller-bottle experiments without added dispersants showed the rapid formation of MOS (Ziervogel et al., 2012; Gutierrez et al., 2013), MOS formation is very likely a biologically driven process. Halomonads, in particular, are commonly linked with the production of large quantities of EPS (Quesada et al., 1994; Béjar et al., 1998; Calvo et al., 1998, 2002; Arias et al., 2003; Gutierrez et al., 2007), and like for many other EPS-producing marine bacteria (e.g., *Alteromonas*, *Pseudoalteromonas*), they can contribute large quantities of EPS to the total DOM pool in the ocean (Azam, 1998). In fact, a large fraction of the DOM in the ocean is of glycoprotein in composition (Long and Azam, 1996; Verdugo et al., 2004), which is consistent with the composition of marine bacterial EPS (Mancuso Nichols et al., 2004; Gutierrez et al., 2007; Hassler et al., 2011). This concurs with our observation of MOS aggregates under the microscope after staining with AB or CBBG, which revealed marine snow formed in the SW+N treatments and MOS formed in the CEWAF and CEWAF+N treatments is largely composed of glycoprotein, and is evidence that it is of biogenic (likely bacterial) origin.

It has been suggested that MOS formation is initiated via the physicochemical interaction between oil droplets, microbial cells and biopolymer – the latter likely of microbial origin (Passow et al., 2012). Our results showed that the presence of a dispersant (Superdispersant-25) enhances MOS formation, as observed in the CEWAF treatment and reported elsewhere using the dispersant Corexit that was used at DWH (Fu et al., 2014). Nutrients were, however, found to amplify the abundance and size of MOS, as observed in the CEWAF+N treatment. However, the fact that marine snow was formed in the SW+N treatment, without any added dispersant, suggests that MOS formation is indeed a biologically driven process that likely involves endogenous DOM in seawater (in the form of TEP and EPS) and which is likely enhanced by the *de novo* synthesis of EPS by EPS-producing bacteria. This is supported by the diversity of EPS-producing taxa we identified enriched on MOS that formed in the CEWAF+N treatment. Correlating this to the Gulf of Mexico environment where profuse quantities of MOS were observed during the DWH oil spill, Lin and Guo (2015) found elevated levels of dilute-HCL-resistant polysaccharides (HR-PCHO) abundance and total dissolved carbohydrates-to-dissolved organic carbon (TCHO/DOC) ratio at some sampling stations. This likely resulted from enhanced microbial production of EPS due to the presence of oil components and nutrient inputs from the Mississippi river (Muschenheim and Lee, 2002; Khelifa et al., 2005).

The CEWAF and CEWAF+N treatments simulated the application of a UK-approved dispersant (Superdispersant-25) and of nutrients – approaches that are often used as a bioremediation strategy for combatting marine oil spills – to investigate the microbial response in surface waters of the FSC. Although we did not conduct measurements for nutrient concentrations, the observed increase in prokaryotic cell abundance, as well as significantly greater α- and β-diversities, in the SW+N treatments is indicative that nutrients are a significant limiting factor in surface waters of the FSC. In support of this, experimental studies in the North Atlantic have shown that bacterial growth can be restricted by the availability of PO_4_^3-^ (Cotner et al., 1997; Rivkin and Anderson, 1997; Caron et al., 2000). Interestingly, Kleindienst et al. (2015b) observed highest prokaryotic cell abundances in WAF treatments by the end of their experiments, whereas we reported highest abundances in the CEWAF+N treatments. This difference across these two studies may be explained by differences in endogenous concentrations of nutrients in the Gulf of Mexico compared to in the FSC that could support growth without addition of an exogenous carbon source (e.g., crude oil or dispersant). Differences in crude oil constituents and their solubility, as well as concentrations of labile/semi-labile DOM between these studies should also be considered. We also measured higher cell abundances in the CEWAF treatments compared to in the WAF treatments. Taken collectively, these results suggest that the presence of dispersant, and particularly added nutrients, stimulate microbial growth in FSC surface waters when contaminated with crude oil. Whether any microbial group was able to degrade and grow on the dispersant used in this study (Superdispersant-25) remains to be investigated.

Our microbial community analysis of FSC surface waters indicated that members of the order *Alteromonadales* and *Rhodobacterales* constituted the dominant proportion (96%) of total sequence reads – lineages which are consistently found and often in high relative abundance in surface waters of the Gulf of Mexico (Yang et al., 2016) and open-ocean Atlantic (Swan et al., 2011). However, the exception was a lack of representation by the SAR11 clade, which is a major group that is commonly found in pelagic waters (Morris et al., 2002) and, quite possibly, because this group has been shown to be susceptible to oil pollution (Lanfranconi et al., 2010; Chronopoulou et al., 2015). Whilst we are planning to analyze whether the surface waters of the FSC are contaminated with hydrocarbons, as might be likely due to the prevalent oil extraction activities occurring in these waters and in those of the adjacent North Sea, the presence of hydrocarbon contaminants could explain the undetectable presence of these organisms in our sequencing libraries. *Colwellia* is a genus of psychrophilic marine heterotrophic generalists, which expectedly was found in the cold surface waters of the FSC, but atypically in quite high relative abundance. Unlike the rapid colonization of these organisms in sea surface oil slicks and subsurface oil plume in the Gulf of Mexico during the DWH spill (Redmond and Valentine, 2012; Yang et al., 2016), the dramatic decline of *Colwellia* in our experiments amended with dispersant, crude oil or both suggests that these organisms may too be susceptible to hydrocarbons in FSC surface waters and to synthetic dispersants, such as Superdispersant-25. Their rapid reduction in the SW+N treatment, however, suggests that these organisms may also suffer a competitive disadvantage to other members of the community during periods of spiked nutrient influxes. *Colwellia* in the surface waters of the FSC may be physiologically inclined as strict oligotrophs. This in contrast to certain oligotypes of *Colwellia* that were identified in the Gulf of Mexico with a predilection for degrading and growing on the dispersant Corexit and crude oil (Kleindienst et al., 2015b). Of further interest, surface waters of the FSC contained a dominance of *Sulfitobacter* (up to 28%), which is a sulfite-oxidizing member of the *Alphaproteobacteria* within the *Roseobacter* clade (Buchan et al., 2005). The abundance of these organisms dramatically fell and was sustained at low levels (often < 2%) by the presence of either exogenous nutrients or crude oil. However, in the presence of dispersant (+/- crude oil and nutrients), an initial dramatic reduction in their abundance was followed by their recovery to abundances >5% and as high as 50%. Since Superdispersant-25 is a sulfur-containing dispersant, it is likely that certain members of the *Sulfitobacter* community sustained a relative high abundance in these dispersant-amended treatments because they were capable of feeding on the sulfur constituent as an energy source.

The presence, albeit in relative low abundances (<0.6%), of obligate hydrocarbonoclastic bacteria (*Alcanivorax*, *Cycloclasticus*, *Oleispira*, *Thalassolituus*) – organisms that are recognized as key players in the biodegradation of crude oil and its refined petrochemical products in the marine environment (Yakimov et al., 2007) – was not unexpected, and included representation of the ‘generalist’ oil-degrader *Marinobacter*. These organisms are typically found at background levels in the global ocean (Yakimov et al., 2007). With the exception of *Oleispira*, the intermittent (1 week) or sustained (over several weeks) bloom of these organisms in the CEWAF and/or CEWAF+N treatments is reminiscent of their strong enrichment in oil-impacted environments where they can be expected to increase in numbers from near undetectable levels. Other taxa that were also strongly selected for in these treatments included *Halomonas*, *Alteromonas* and *Pseudoalteromonas* – genera that contain members with reported hydrocarbon-degrading capabilities, though are more commonly associated with producing EPS (Béjar et al., 1998; Calvo et al., 1998, 2002; Arias et al., 2003; Mancuso Nichols et al., 2004; Bhaskar and Bhosle, 2005; Gutierrez et al., 2007, 2008, 2009, 2013). Interestingly, a study that investigated the response of pelagic bacterial communities to crude oil in the North Sea showed that the most dominant responder was *Pseudoalteromonas* (10-fold enrichment), with practically no detection for any of the obligate hydrocarbonoclastic taxa; however, denaturing gradient gel electrophoresis (DGGE) of the bacterial 16S rRNA gene was used for analyzing microbial community profiles in this study which, based on its limited coverage for capturing near total diversity, will likely have missed less abundant taxa (Chronopoulou et al., 2015). Nonetheless, this highlights how different water bodies, even those adjacent to each other at the same or proximal latitude, can yield differential microbial community responses to crude oil contamination, which may be attributed to, though not always entirely, to a predilection of certain taxa to hydrocarbons.

Interestingly, a short, but strong enrichment in the CEWAF and CEWAF+N treatments for members of the *Vibrionales* – principally the genus *Vibrio* – revealed that these organisms may participate in the degradation of crude oil in FSC surface waters. The enrichment of these organisms is not frequently observed at contaminated sites in the marine environment, although there are snippets in the literature reporting on the enrichment of these organisms by crude oil. For example, members of the *Vibrionales* were found enriched in beach sands of the Gulf coast that had become contaminated with Macondo oil from the DWH spill, and several oil-degrading *Vibrio* spp. were isolated and found to degrade hydrocarbons (Kostka et al., 2011). Also, a 91-fold increase in the relative abundance of *Vibrionales* was detected in oil contaminated sea surface oil-slick water samples from DWH when incubated to develop anaerobically (Gutierrez et al., 2016). An analysis of the genomes of several *Vibrio* species found these organism capable of metabolizing hydrocarbons, including PAHs (Grimes et al., 2009). Further work will be needed to fully understand the hydrocarbon-degrading potential and role of these organisms in the FSC.

This study highlights the importance for the application of dispersants and/or nutrient-amendments in MOS formation in the event of an oil spill in the FSC. We also identified oil-degrading and EPS-producing bacteria associated with MOS, and that crude oil alone does not yield MOS in these waters. We note that the seawater used in this study was obtained from one seasonal period of the year (the winter of 2015) and that further work would be needed to explore MOS formation in waters collected during other seasons in order to provide a more conceptual understanding of this process given the unpredictability of when an oil spill might occur in the FSC. We also demonstrate that surface waters of the FSC harbor communities of hydrocarbonoclastic bacteria that positively respond to crude oil contamination, and that amending these waters with dispersant and/or nutrients could stimulate microbial community activities. Based on our findings, such approaches should be considered in bioremediation strategies in the event of a major oil spill in this region of the northeast Atlantic, although further instigative work to assess this is warranted. Essentially, the influence of dispersants on oil-degrading bacteria remains poorly understood and requires further investigation using different types of dispersants and evaluation across different water bodies. Our findings on MOS formation and the microbial response to oil in FSC surface waters mirror those observed at DWH and hence underscore their broad relevance.

## Conclusion

Marine oil snow is possibly the most important mechanism by which oil reaches the seafloor in the event of a spill at sea. Our study shows that in the event of an oil spill in the FSC, the use of dispersants would likely lead to the formation of MOS and trigger a subsurface “dirty blizzard,” reminiscent to that during the DWH oil spill where a large proportion of sea surface oil ended up on the sea floor. In the absence of dispersant applications, the majority of surface oil is likely to remain at the sea surface. Hence, any research conducted to evaluate crude oil impacts to benthic ecosystems in the FSC would need to take into account the physicochemical state of the oil presented in the form of MOS aggregates – direct exposure of sediment samples or cores to crude oil for such investigations would be unrealistic. Our study also showed that MOS particles formed with FSC surface seawater harbor rich communities of prokaryotes, including oil-degrading bacteria, potentially acting as ‘hot spots’ where a heightened level of oil biodegradation occurs.

## Author Contributions

LDS and TG contributed to the design of the work and its interpretation. LDS and SS produced all of the data and, together with TG, wrote the manuscript.

## Conflict of Interest Statement

The authors declare that the research was conducted in the absence of any commercial or financial relationships that could be construed as a potential conflict of interest.

## Footnotes

1 https://www.liverpool.ac.uk/genomic-research

## Supplementary Material

FIGURE S1 **Rarefaction curves signify the α-diversity of all treatments across all sampling times**. OTUs are identified based on similiarity of the 16S rRNA gene (97%). SW, seawater; SW+N, seawater with nutrients; SW+D, seawater with dispersant; WAF, water-accomodated fraction; CEWAF, chemically enhanced WAF; CEWAF+N, chemically enhanced WAF with nutrients. Sample points are: T_1_, **(A)**; T_2_, **(B)**; T_4_, **(C)**; T_6_ **(D)**.

FIGURE S2 **The Shannon-Weiner diversity indices are presented here as a boxplot comparing the α-diversity between water treatments **(A)** and time points **(B)** measured in this study**.

TABLE S1 **Composition of MiSeq sequenced 16S rRNA gene reads, analyzed in genus-, family-, and order-level resolution for each of the different treatments**.
